# Supplementary material for: New Strategies to Increase the Abiotic Stress Tolerance in Woody Ornamental Plants in Mediterranean Climate
Source: Plants (Basel). 2023 May 18;12(10):2022. doi: 10.3390/plants12102022 (PMC10223706; doi:10.3390/plants12102022)
Supplement: Supplementary file 1 [file plants-12-02022-s001.zip › plants-2372128-supplementary.pdf]

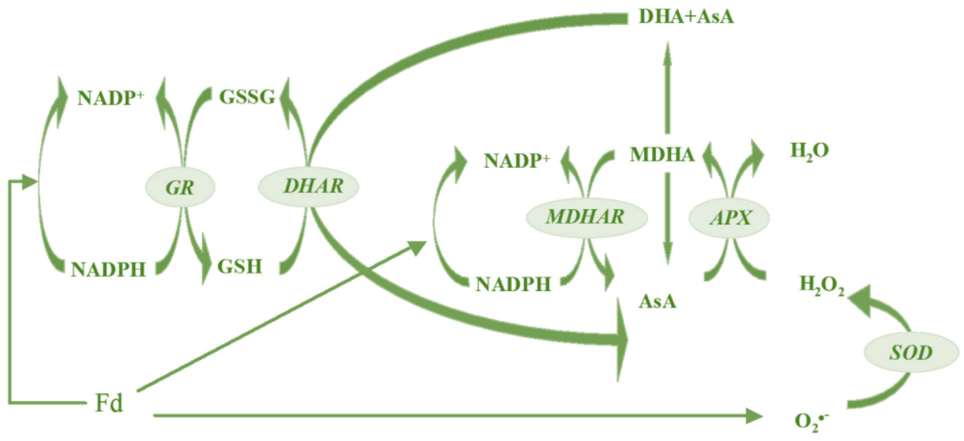

Figure S1. The detoxification enzymes belonging to ascorbate-glutathione cycle. The superoxide radicals ($O_2^{\cdot-}$) are removed by superoxide dismutase (SOD) which leads to hydrogen peroxidase (H_2O_2) formation. The ascorbate (AsA) is regenerated by oxidized forms by a cycle catalyzed by ascorbate peroxidase (APX) enzyme. The AsA is covered in monodehy-droascorbate (MDHA) by APX, then the monodehyroascorbate reductase (MDHAR) oxides the MDHA and reduces the $NADP^+$ that is regenerated by Ferredoxin (Fd). The MDHA can be converted to ASA and dehydroascorbate (DHA) by non-enzymatic reaction. The DHA is converted by dehydroascorbate reductase enzyme (DHAR) in ASA, contemporary the reduced glutathione (GSH) is transformed in oxidized glutathione (GSSG) by NADPH.
